# Sodium 4-phenylbutyrate suppresses the development of dextran sulfate sodium-induced colitis in mice

**DOI:** 10.3892/etm.2013.1456

**Published:** 2013-12-19

**Authors:** KAZUHIKO ONO, SATOSHI NIMURA, TAKUYA NISHINAKAGAWA, YUKO HIDESHIMA, MUNECHIKA ENJYOJI, KAZUKI NABESHIMA, MANABU NAKASHIMA

**Affiliations:** 1 Department of Immunological and Molecular Pharmacology, Faculty of Pharmaceutical Science, Jonan-ku, Fukuoka 814-0180, Japan; 2 Department of Pathology, Faculty of Medicine, Fukuoka University, Jonan-ku, Fukuoka 814-0180, Japan

**Keywords:** phenylbutyrate, dextran sulfate sodium, inflammatory bowel disease

## Abstract

Sodium 4-phenylbutyrate (PBA) exhibits anti-inflammatory effects by suppressing nuclear factor-κB (NF-κB) activation. In the present study, the effects of PBA on a mouse model of dextran sulfate sodium (DSS)-induced colitis were investigated. The therapeutic efficacy of PBA (150 mg/kg body weight) in DSS-induced colitis was assessed based on the disease activity index (DAI), colon length, the production of inflammatory cytokines and histopathological examination. The results showed an increase in the median survival time in the PBA-treated group compared with that of the untreated DSS control group. DAI scores were lower in the PBA-treated group than in the DSS control group during the 12 days of the experiment. Additionally, PBA treatment inhibited shortening of the colon and the production of the inflammatory cytokines tumor necrosis factor-α, interleukin-1β and IL-6, which were measured in the colonic lavage fluids. Histopathological examination of the DSS control group showed diffused clusters of chronic inflammatory cells infiltrating the lamina propria, partial exfoliation of the surface epithelium and decreased numbers of mature goblet cells. By contrast, in the PBA-treated group the histopathological findings were the same as those of the normal healthy controls. These results suggest that PBA strongly prevents DSS-induced colitis by suppressing the mechanisms involved in its pathogenesis.

## Introduction

Although the inflammatory reaction is vital to homeostatic maintenance, it may also trigger or further aggravate various diseases, including inflammatory bowel disease (IBD), which is a group of chronic inflammatory disorders of the gastrointestinal tract. The hallmark of IBD is chronic, uncontrolled inflammation involving any section of the gastrointestinal tract in Crohn’s disease and limited to the colon in ulcerative colitis. Both diseases are characterized by weight loss, fever, gastric dysmotility, colonic mucosal ulceration, shortening of the colon and diarrhea with blood and/or mucus. These characteristics are exacerbated by immunological disorders that activate cellular and humoral immune responses, particularly those involving neutrophils (1–4). Although a number of studies have indicated roles for genetic, environmental and lifestyle effects in the pathogenesis of IBD, its etiology remains poorly understood (5–9). Patients with IBD are typically treated with agents that target aberrant immune responses and inflammatory cascades, including anti-inflammatory agents (5-aminosalicylic acid and glucocorticosteroids), immunomodulatory therapy (azathioprine, 6-mercaptopurines and cyclosporine) and monoclonal antibody therapy (for example, with anti-TNF-α and leukapheresis) (10–14).

Sodium 4-phenylbutyrate (PBA) is a phenyl-substituted short-chain fatty acid used in the treatment of a wide range of diseases, such as urea metabolism disorders (15), homozygous β-thalassemia (16), spinal muscular atrophy (17) and tumors (18). In recent studies, PBA was shown to suppress oxidative stress by attenuating endoplasmic reticulum stress and to exert anti-inflammatory effects by suppressing the activity of the transcriptional factor, nuclear factor-κB (NF-κB) (19–22). Furthermore, we previously identified that PBA may be effective in the treatment of neurodegenerative diseases, including Parkinson’s disease (23).

In the present study, the effects of PBA were investigated on colonic inflammation in a mouse model of colitis induced by dextran sulfate sodium (DSS), which is a standard animal model of IBD.

## Materials and methods

### Experimental animals

Male ICR mice (weight, 28–30 g) were purchased from Kyudo, Co., Ltd. (Saga, Japan). All mice were housed in cages (n=5 per cage) with a 12 h light:dark cycle and a constant temperature of 20±5°C. The animals (n=45) were divided into the following groups: The normal control group (n=5), DSS-treated control group (n=10) and groups treated with DSS plus 50 (n=5), 100 (n=10), 150 (n=10) and 200 (n=5) mg PBA/kg body weight. Survival rates, the development of DSS-induced colitis and cytokine levels were analyzed in all groups. An additional 10 mice were treated with DSS and 150 mg PBA/kg body weight, and the colon length and histopathological changes were evaluated on day 8. The animals were fed standard laboratory chow and had access to water *ad libitum*. All animal experiments were conducted under the University guidelines and were approved by the Ethical Committee for Animal Care and Use of Fukuoka University (Fukuoka, Japan).

### DSS and PBA treatment

Experimental colitis was induced by the addition of 3.5% (w/v) DSS (Nacalai Tesque, Inc., Kyoto, Japan) to the drinking water of the mice. PBA (LKT Laboratories Inc., St. Paul, MN, USA) was administered via intraperitoneal injection at doses of 50, 100, 150 or 200 mg PBA/kg body weight on days 0, 2, 4, 6, 8, 10 and 12.

### Assessment of DSS-induced colitis

DSS-induced colitis was characterized by the presence of acute colitis, bloody mucoid stools, diarrhea, abdominal pain, weight loss, shortening of the colon and mucosal ulceration with neutrophil infiltration (24). Mice with DSS-induced colitis were evaluated using a disease activity index (DAI), which assigns a score to changes in weight, a positive Hemoccult test, gross blood in the stools and stool consistency. Body weight, stool consistency and the condition of the peri-anal tissues were recorded daily. The presence of fecal occult blood was tested by collecting colonic lavage fluid immediately prior to the intraperitoneal injection of PBA. The DAI was then scored for three categories as follows: Body weight loss (0, none; 1, 1–5%; 2, 5–10%; 3, 10–20%; and 4, >20%), stool consistency (0, normal; 2, loose stools; and 4, diarrhea), and stool blood (0, negative; 2, positive Hemoccult test; and 4, gross bleeding). Body weight loss was calculated as the percentage difference between the body weight on day 0 and that on the day the animal was weighed. For mortalities during this study, the last determined DAI score was used and mice that accidentally died during the experiment were excluded (25).

### Measurement of cytokines by enzyme-linked immunosorbent assay (ELISA)

Cytokines in the collected colonic lavage fluid were measured by ELISA. Briefly, 96-well plates were coated with monoclonal antibodies [2.0 μg/ml anti-mouse tumor necrosis factor-α (TNF-α), purified; 2.0 μg/ml anti-mouse/rat interleukin-1β (IL-1β), purified; and 1.0 μg/ml anti-mouse IL-6, purified] overnight at 4°C and washed with phosphate-buffered saline (PBS). Blocking One solution (Nacalai Tesque, Inc.) was diluted 5-fold with PBS and added to the plates. After 1 h of incubation at room temperature (RT), the collected colonic lavage fluid was added to the plates in a 1:10 dilution. The plates were further incubated for 1 h at RT and then washed with PBS. Biotinylated antibodies (0.8 μg/ml biotinylated anti-mouse TNF-α; 2.0 μg/ml biotinylated anti-mouse/rat IL-1β; and 1.0 μg/ml biotinylated anti-mouse IL-6) were added and the plates were incubated for 1 h at RT. Streptavidin horseradish peroxidase (SNN 1004; Biosource International, Inc., Camarillo, CA, USA; 1:10,000) was then added and the plates were incubated for 1 h at RT. After the plates had been washed thoroughly, 100 μl peroxidase substrate solution consisting of equal volumes of 3,3′,5,5′-tetramethylbenzidine (TMB) peroxidase substrate and peroxidase substrate solution B (TMB Microwell Peroxidase Substrate System; KPL Inc., Gaithersburg, MD, USA) were added to each well for 10 min, followed by an equal volume of stop solution. Absorbance was measured at 490 nm with an ELISA reader (Bio-Rad Model 450 Microplate Reader; Bio-Rad, Hercules, CA, USA). All anti-cytokine antibodies were purchased from eBioscience (San Diego, CA, USA).

### Measurement of colon length and histopathological evaluation

On day 8, the 10 mice with DSS-induced colitis that were treated with 150 mg PBA/kg body weight (as described in Experimental animals) were sacrificed and a section of the colon extending from the cecocolic junction to the anus was removed. Colon length was measured between the cecocolic junction and the anal verge. The resected tissue was fixed overnight in 10% neutral-buffered formalin (Nacalai Tesque, Inc.) and then routinely processed for embedding in paraffin blocks. Sections of 3-μm thick tissues were cut and stained with hematoxylin and eosin for examination under a microscope (OLYMPUS BX51; Olympus Corporation, Tokyo, Japan).

### Statistical analysis

**Figure 1:**
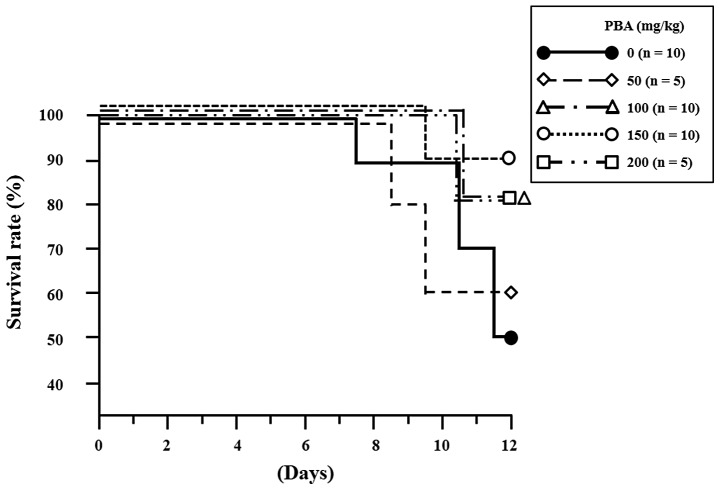
Survival rates of ICR mice receiving 3.5% DSS and varied doses of PBA for 12 days. ^•^DSS control group (n=10), ^⋄^DSS + 50 mg PBA/kg body weight (n=5), ^Δ^DSS + 100 mg PBA/kg body weight (n=10), ^○^DSS + 150 mg PBA/kg body weight (n=10), and ^□^DSS + 200 mg PBA/kg body weight (n=5). DSS, dextran sulfate sodium; PBA, sodium 4-phenylbutyrate.

**Figure 2:**
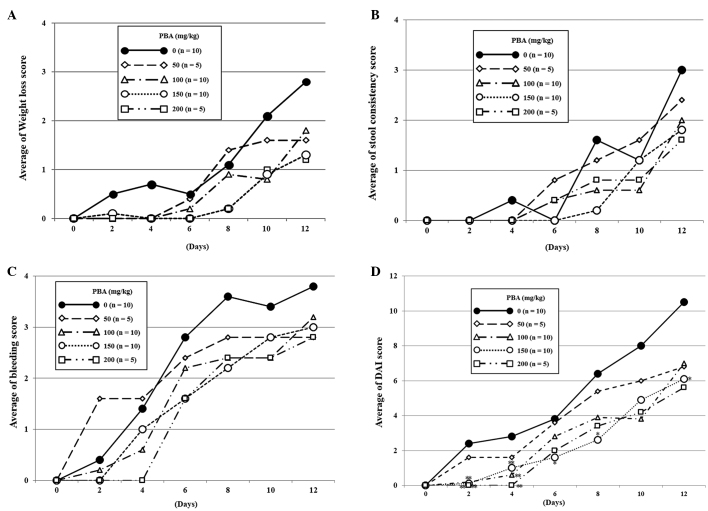
Effects of DSS and/or PBA supplementation on the DAI scores during 12 days of the experiment. Mean (A) weight loss score, (B) stool consistency score, (C) bleeding score, and (D) total DAI score (n=5 or 10 per group). The data are summarized as the means ± standard error. ^*^P<0.05; ^**^P<0.01 compared with the DSS control group, determined by one-way analysis of variance followed by Dunnet’s post hoc test. DSS, dextran sulfate sodium; PBA, sodium 4-phenylbutyrate; DAI, disease activity index.

**Figure 3:**
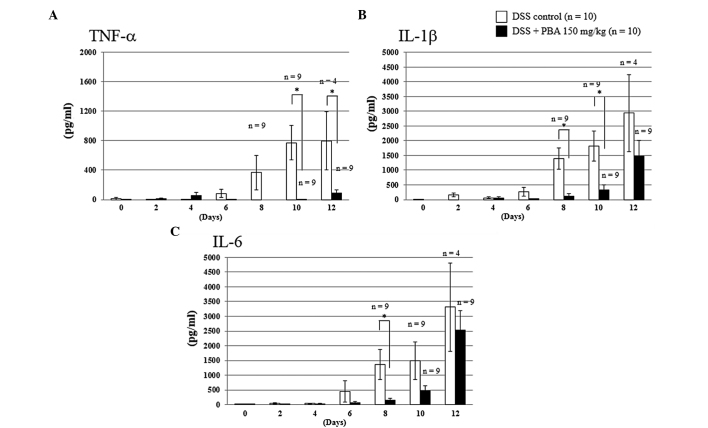
Cytokine levels of (A) TNF-α, (B) IL-1β and (C) IL-6 in the collected colonic lavage fluid. TNF-α, tumor necrosis factor-α; IL, interleukin. ^*^P<0.05; values were analyzed by one way analysis of variance and Student’s t-test.

**Figure 4:**
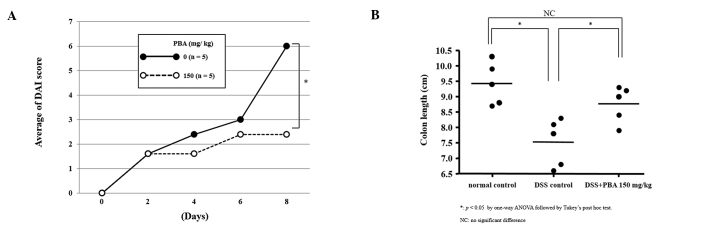
Effects of PBA treatment on colon length on day 8 of the experiment. (A) DAI scores of the DSS control group (n=5) and the 150 mg/kg body weight PBA treated group (n=5). (B) Colon length of the experimental groups. Data are expressed as the mean ± standard error. ^*^P<0.05. Values were analyzed by one-way analysis of variance followed by a Tukey-Kramer post hoc test. PBA, sodium 4-phenylbutyrate; DAI, disease activity index; DSS, dextran sulfate sodium.

The experimental data were statistically analyzed using the log-rank test (Fig. 1), one-way analysis of variance followed by Dunnet’s post hoc test (Fig. 2), Student’s t-test (Figs. 3 and 4A) and the Tukey-Kramer post hoc test (Fig. 4B). P<0.05 was considered to indicate a statistically significant difference. Data are expressed as the mean ± standard error.

## Results

### Survival rate

On day 12, the survival rate of DSS-treated control mice was 50% (5/10), whereas the mice treated with 50, 100, 150 and 200 mg PBA/kg body weight showed survival rates of 60% (3/5), 80% (8/10), 90% (9/10) and 80% (4/5), respectively (Fig. 1). The larger number of animals in the 100 and 150 mg PBA/kg body weight groups reflects an initial test group of five animals and then the testing of the effects of these drug doses in five additional animals. The differences in survival rates from those in the untreated control group were not identified to be significant, possibly due to the small sample size. However, there was a positive trend in the survival rates of the PBA-treated animals. The following results in this study refer to those obtained from the mice treated with 150 mg PBA/kg body weight as this dose yielded the highest survival rate.

### DAI score

In the DSS control group, occult blood positivity was observed on day 2 in two of the 10 mice and on day 4 in the remaining eight mice. Gross bleeding was observed in four mice on day 6. In the mice treated with 150 mg PBA/kg body weight, occult blood positivity was detected on days 4 (5/10) and 6 (8/10), which was later than that of the DSS control group. Only one mouse showed gross bleeding, which was observed on day 6 (Fig. 2C). By the end of the experiment, there were seven cases of severe weight loss (DAI score of ≥3) in the DSS control group, but only one case in the PBA group, which was detected on day 12 (Fig. 2B). Overall, the DAI score of the PBA-treated group was significantly lower than that of the DSS control group on days 2, 4, 6, 8 and 12 (Fig. 2D).

### Inflammatory cytokine production (ELISA)

The concentrations of cytokines in colonic lavage fluid were determined from a standard curve prepared for each plate. In the DSS-treated control group, the level of TNF-α production appeared to increase from day 6, reaching 365.7±232.6 pg/ml by day 8. However, in the PBA group, TNF-α production was suppressed completely until day 8 and only low levels were detected on day 10. Similarly, on day 8 the levels of IL-1β and IL-6 production in the PBA-treated group were suppressed by 91.3% (121.9±72.4 pg/ml) and 88.9% (151.3±68.8 pg/ml), respectively, compared with those of the DSS control group (1,398.0±358.5 and 1,362.8±502.8 pg/ml, respectively). The differences between the DSS-treated control and PBA groups were significant (P<0.01) for all three cytokines (Fig. 3).

### Colon length

The protective effects of PBA against DSS-induced shortening of the colon were examined in five mice on day 8, when the DAI score of the PBA-treated group was significantly lower than that of the DSS control group (Fig. 4A). The animals were sacrificed and the length of the colon from the cecocolic junction to the anal verge was measured. The tissues were then processed for histology. The length of the colon was 9.4±0.3 cm in healthy control animals, 7.5±0.3 cm in the DSS control group and 8.8±0.3 cm in the PBA-treated group. The difference between the latter two groups was statistically significant, indicating marked suppression of the DSS-induced shortening of the colon in mice treated with 150 mg PBA/kg body weight (Fig. 4B).

### Histological findings

**Figure 5:**
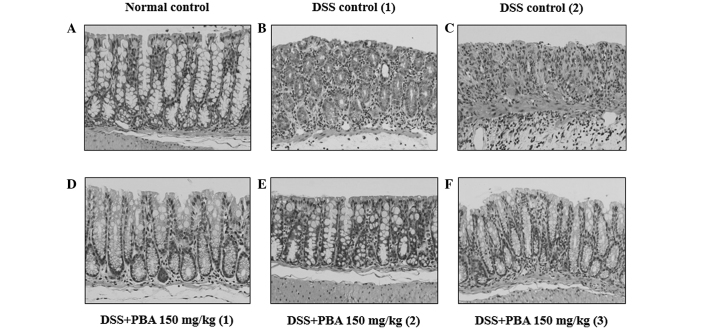
(A) Low-power view of a longitudinal section of the normal colonic wall. Crypts with abundant goblet cells were observed. (B) Chronic inflammation in the lamina propria of mice with DSS-induced colitis was observed. The loss of goblet cells and the frequently enlarged nuclei of the absorptive cells was observed. (C) Unequivocal mucosal erosion appeared due to the loss of surface epithelia. (D–F) PBA-treated mice showed no abnormalities. DSS, dextran sulfate sodium; PBA, sodium 4-phenylbutyrate. Magnification, ×200.

Microscopic examination of the excised colon segments in the DSS control group showed chronic inflammatory cells infiltrating the lamina propria around the crypts. The surface epithelia were partially exfoliated, revealing the underlying intestinal mucosa. The epithelium did not contain mucin of a mature goblet cell and thus indicated mucin depletion (Fig. 5B and C). By contrast, in the PBA-treated group the mucosa showed a normal appearance and resembled that of healthy animals (Fig. 5A, D–F).

## Discussion

In the present study, the effects of PBA were investigated on DSS-induced colitis in mice as a model of IBD. DSS-treated mice tested positive for occult blood and loose stools were observed as early as day 2. Evident weight loss developed by day 6, and by day 10, five of the 10 mice had died. However, in DSS mice treated with 150 mg PBA/kg body weight, positive occult blood was first detected on day 4 and loose stools and weight loss did not occur until day 8. By day 10 there was only one mortality (Fig. 2A–D). Thus, by day 12, mice in the PBA-treated group had a 40% better survival rate than those of the DSS control group (Fig. 1), which is consistent with the lower DAI score (an index of clinical state) in the PBA-treated group. PBA also significantly restricted DSS-induced shortening of the colon (Fig. 4B) and maintained mucosal integrity to the extent of that observed in the healthy control group (Fig. 5A,D–F). These results suggest that PBA treatment suppressed or limited the development of DSS-induced colitis, histological inflammation and shortening of the colon.

Previous studies have demonstrated the pivotal role of abnormally high levels of pro-inflammatory cytokines, such as TNF-α, IL-1β and IL-6, within colonic tissues in the pathogenesis of IBD. Thus, blocking the production of these inflammatory mediators may be of therapeutic potential in patients with IBD (26–28). Notably, promising results have been obtained in patients with IBD who were treated with anti-TNF-α therapy (12,29,30). Agents targeting interferon-γ or the IL-6 receptor are currently undergoing clinical trials (31). However, as with most biological therapeutics, the risk of developing immunogenic side-effects, such as infusion reactions and serum sickness-like reactions, are of major concern, particularly in patients treated for chronic diseases. Small-molecule therapeutics, including PBA, are devoid of these side-effects and have the additional benefit of an oral route of administration (32,33). In the present study, the concentrations of TNF-α, IL-1β and IL-6, which were measured in the colonic lavage fluids, increased in parallel with worsening of the disease state in the DSS control group, whereas this was not the case in the PBA-treated group. PBA treatment almost completely suppressed TNF-α production until the end of the experiment. However, while IL-1β and IL-6 production were suppressed by PBA during the early disease phase, by the end of the experiment the levels of both cytokines had increased and did not differ significantly from those of the DSS-induced colitis group. However, this early suppression was sufficient to inhibit the onset of DSS-induced colitis, as indicated by the DAI, the histopathological findings and the improved survival rate of the PBA-treated mice.

The etiology of IBD remains poorly understood and affected patients are only treated symptomatically. Moreover, the few medications that have been demonstrated to be effective are often associated with considerable adverse side-effects, which further complicates the medical therapy of IBD. In the present study, PBA treatment was shown to suppress the onset of DSS-induced colitis. While the efficacy of PBA appears to involve the suppression of pro-inflammatory cytokines, further studies are required to elucidate the precise mechanisms of action. The findings of these studies may ultimately allow the clinical use of PBA to improve the pathological conditions and disease remission rates of patients with IBD.
